# Supplementary material for: CO2 fixation by anaerobic non-photosynthetic mixotrophy for improved carbon conversion
Source: Nat Commun. 2016 Sep 30;7:12800. doi: 10.1038/ncomms12800 (PMC5056431; doi:10.1038/ncomms12800)
Supplement: Supplementary Information — Supplementary Figures 1-3, Supplementary Tables 1-3 [file ncomms12800-s1.pdf]

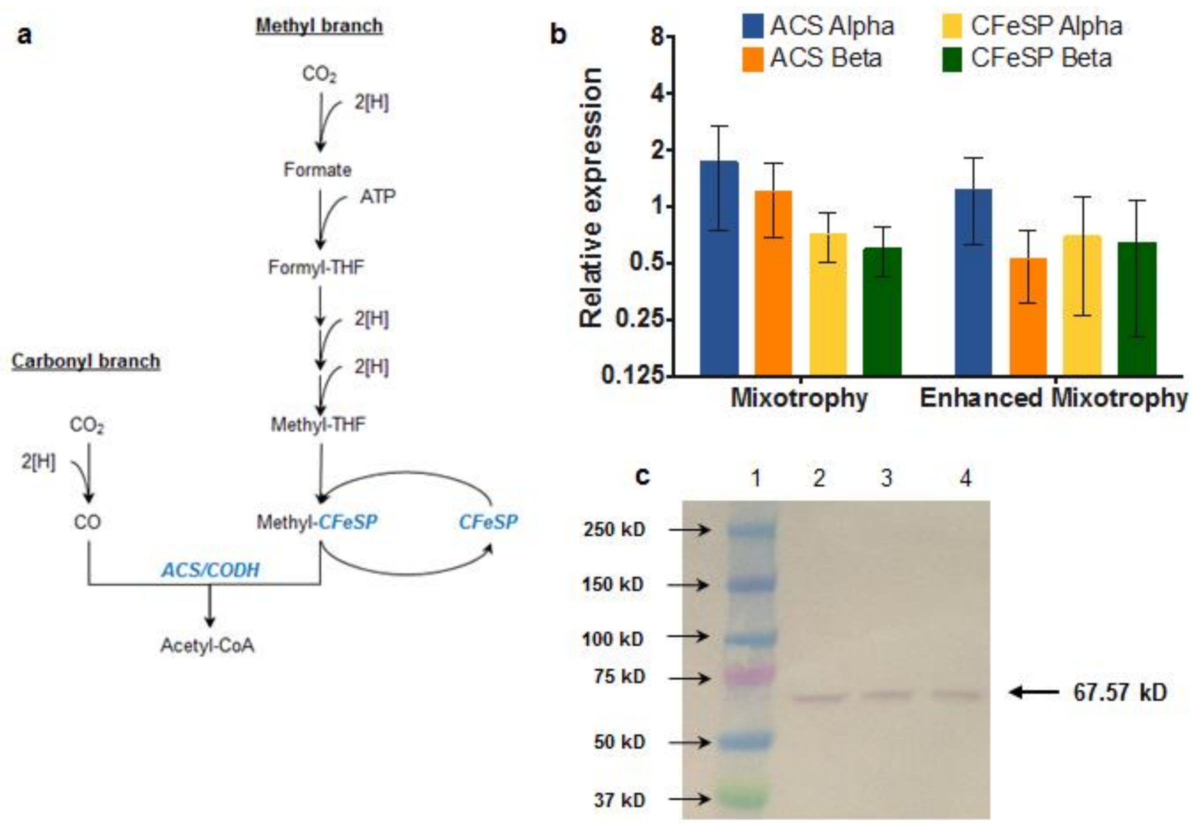

Supplementary Figure 1. qRT-PCR and Western blots for CLJ cultures under autotrophic and mixotrophic conditions. a, Schematic of the WLP with the genes/enzymes assayed in blue italics. **b**, qRT-PCR results for four genes of the WLP. The four genes assayed were two genes encoding for the corrinoid iron-sulfur protein (CFeSP, c37570 and c37580) and two genes encoding for the acetyl-CoA synthase (ACS, c37550 and c37670). The relative expression of each gene was determined compared to the autotrophic control. Expression values <1.0 indicate a lower expression in mixotrophy or enhanced mixotrophy compared to autotrophy, and expression values >1.0 indicate a higher expression in mixotrophy or enhanced mixotrophy compared to autotrophy. None of the genes showed statistically significant differences compared to the autotrophic control. **c**, Western blot using polyclonal antibody raised in rabbit against the carbon monoxide dehydrogenase (CODH) subunit of the ACS/CODH. (1) Kaleidoscope™ Ladder (Bio-Rad), (2) CLJ mixotrophy, (3) CLJ autotrophy, and (4) CLJ syngas-enhanced mixotrophy.

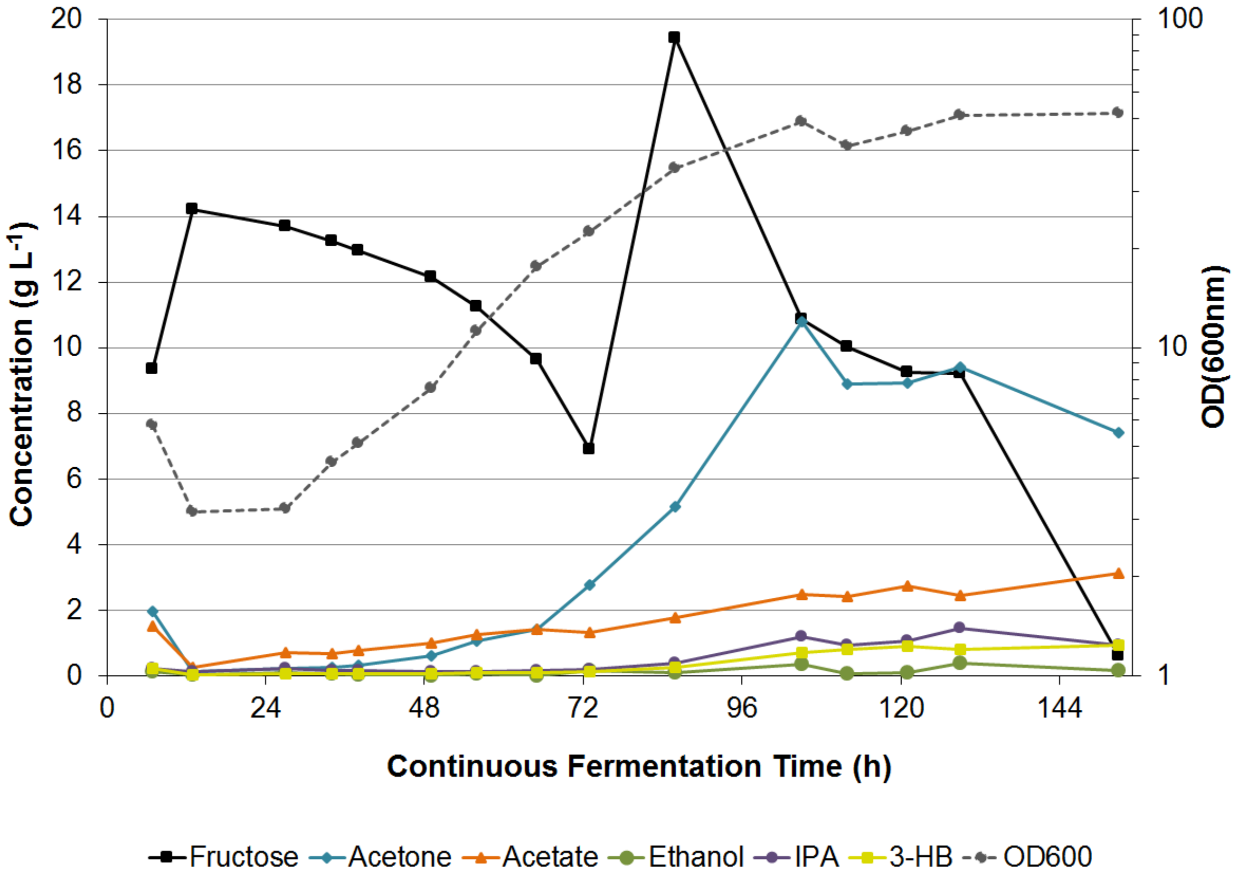

Supplementary Figure 2. High cell density continuous acetone fermentation profiles. This data corresponds to the data presented in Figure 3. Residual fructose concentration (black square), acetone (blue diamonds), acetate (orange triangles), ethanol (green circles), IPA (purple circles), 3-HB (yellow squares), and cell density (OD_{600nm} – grey circles with broken line).

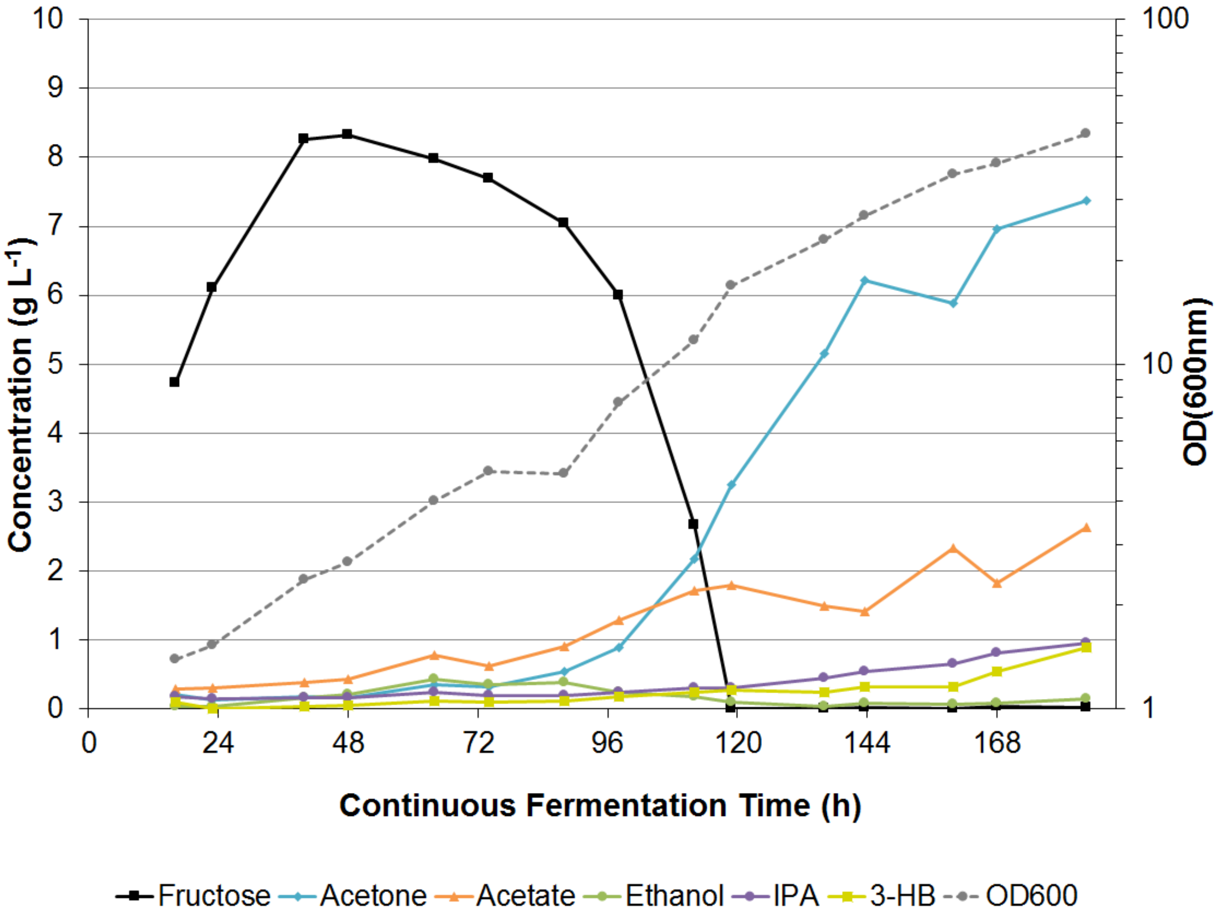

Supplementary Figure 3. Biological replicate high cell density continuous acetone fermentation profiles. Fermentation profiles of a biological replicate high cell density continuous fermentation. Residual fructose concentration (black square), acetone (blue diamonds), acetate (orange triangles), ethanol (green circles), IPA (purple circles), 3-HB (yellow squares), and cell density (OD_{600nm} – grey circles with broken line).

Supplementary Table 1. Product concentrations from Fig. 2c.

**0%(v v^{-1}) H_2**								
				Concentration (g L^{-1})				
Hour	OD_{600nm}	Fructose	Acetone	Acetate	3-HB	Ethanol	2,3-BD	Lactate
0	0.12	4.94	0.08	0.15	0.01	0.52	0.00	0.00
168	1.17	0.00	1.73	1.54	0.29	0.29	0.00	0.01
**20%(v v^{-1}) H_2**								
				Concentration (g L^{-1})				
Hour	OD_{600nm}	Fructose	Acetone	Acetate	3-HB	Ethanol	2,3-BD	Lactate
0	0.12	5.17	0.08	0.17	0.01	0.57	0.00	0.00
168	0.97	0.00	1.02	0.49	0.34	2.42	0.03	0.04
**40%(v v^{-1}) H_2**								
				Concentration (g L^{-1})				
Hour	OD_{600nm}	Fructose	Acetone	Acetate	3-HB	Ethanol	2,3-BD	Lactate
0	0.12	4.74	0.08	0.17	0.01	0.52	0.00	0.00
168	0.57	0.90	0.81	0.48	0.28	1.96	0.02	0.04

Supplementary Table 2. NAD(P)H:Acetyl-CoA ratios for different metabolites.

	Acetate	Butyrate	Ethanol	n-Butanol	Acetone	Isopropanol	Isobutyrate	Isobutanol	Propionate	2,3-BD[1]	1,4-BD[1]	3-HB[1]	2-HIB[1]
NAD(P)H: Acetyl-CoA ratio	0	1	2	2	0	0.5	1	2	3	1.5	1.5 or 7[2]	0.5	0.5
Increase in yield of mixotrophy over heterotrophy	53%	22%	2%	2%	53%	36%	22%	2%	0%	11%	14%	36%	36%

[1] 2,3-BD = 2,3-butanediol; 1,4-BD = 1,4-butanediol; 3-HB = 3-hydroxybutyrate; 2-HIB = 2-hydroxyisobutyrate

[2] 1,4-BD can be produced either through the carboxylation of pyruvate or through the rearrangement of two pyruvate molecules. Thus, the NAD(P)H/Acetyl-CoA ratio can be written as either 1.5 or 7. Given that the WLP can fix carbon to produce one molecule of acetyl-CoA from the four molecules of NAD(P)H, 1.5 and 7 are equivalent numbers in our system.

Supplementary Table 3. PCR primers.

Primer name	Sequence	Purpose
SADH 5’ region For	AGCTGTAGATAACAATGGGATCAT	Amplify 5’ region of homology
SADH 5’ region Rev NotI	TGTAACTACTGAGCGGCCGCGCAAATGTTGTGCCAGTATGT	Amplify 5’ region of homology
SADH 3’ region For NotI	CACAACATTTGCGCGGCCGCTCAGTAGTTACATTCTAAAAATTCATATAAAA	Amplify 3’ region of homology
SADH 3’ region Rev	ACATTGCCTGATTTGAGTTCATC	Amplify 3’ region of homology
pta P For SbfI	TACCTGCAGGAAATGCCTAAGTGAAATATATACATATTAT	Amplify P_{pta}
pta P Rev	TTCTCTCATGGATCCGTTCCTCCCTTTAAATTTAACACAAAATTAC	Amplify P_{pta}
thl CKL For	GGGAGGAACGGATCCATGAGAGAAGTAGTTATTGTAAGTGCAG	Amplify *thl*
thl CKL Rev	CCTCCTCTCGCCGGCTTATCTCTCTACTATTAAAGCAGTTCCCAT	Amplify *thl*
ctfAB CAC For	GAGAGATAAGCCGGCGAGAGGAGGGATTAAAATGAACTCTAAAATAAT	Amplify *ctfAB*
ctfAB CAC Rev	CACCTTCCTCTCGAGCTAAACAGCCATGGGTCTAAGTTC	Amplify *ctfAB*
adc CAC For	GCTGTTTAGCTCGAGAGGAAGGTGACTTTTATGTTAAAGGATG	Amplify *adc*
adc CAC Rev KasI	TGATGCGGCGCCTTACTTAAGATAATCATATATAACTTCAGC	Amplify *adc*
c37550 RT F	GGACTTGACCTCAGGGTTATTC	qRT-PCR for ACS Alpha
c37550 RT R	TGCACCTGCTGAAACTACAA	qRT-PCR for ACS Alpha
c37670 RT F	GGGTCGAAACAGTTTGGGATAG	qRT-PCR for ACS Beta
c37670 RT R	GTACCTGCTGCAACCATTCT	qRT-PCR for ACS Beta
c37570 RT F	TGCAAGAGCACTTCCTCTTTAT	qRT-PCR for CFeSP Alpha
c37570 RT R	CCTGCATCTGGTATAACCATCC	qRT-PCR for CFeSP Alpha
c37580 RT F	GTAGGAGCTGCAGGTGTAATG	qRT-PCR for CFeSP Beta
c37580 RT R	GCTGCAAGCTTTACTCTGTCTA	qRT-PCR for CFeSP Beta
c12520 RT F	AGGTACAGTTTGCAAGGCA	qRT-PCR housekeeping gene
c12520 RT R	ACTTGAGACTTAGGTGTACCATAATAG	qRT-PCR housekeeping gene
